# Regulation of a catabolic enzyme whose substrate is always available

**DOI:** 10.1128/msystems.00369-26

**Published:** 2026-05-26

**Authors:** Oliver Lenz

**Affiliations:** 1 Institute of Chemistry, Biophysical Chemistry, Technische Universität Berlin 26524, Berlin, Germany; Danmarks Tekniske Universitet, Kgs. Lyngby, Lyngby-Taarbæk, Denmark

**Keywords:** *Mycobacterium smegmatis*, high-affinity hydrogenase, carbon catabolite repression

## Abstract

Certain hydrogenases are capable of converting the trace amounts of molecular hydrogen constantly present in Earth’s atmosphere to supply cells with a little energy during periods of starvation. Recently, Kropp and colleagues investigated the regulation of one of these “high-affinity” hydrogenases in response to different growth conditions for the ubiquitous soil bacterium *Mycobacterium smegmatis* (A. Kropp, J. D. Archer, M. Jespersen, T. D. Watts, et al., mSystems 11:e01678-25, 2026, https://doi.org/10.1128/msystems.01678-25). A mutation in the *gylR* gene, whose product acts as a positive transcriptional regulator of the genes involved in glycerol catabolism, causes the corresponding mutant strain to grow very slowly on glycerol, while the activity of the high-affinity hydrogenase reached levels more than 50 times higher than those of the wild-type strain of *M. smegmatis*. The results of this study suggest that the synthesis of the hydrogenase is subject to a regulatory mechanism similar to carbon catabolite repression, which is entirely consistent with the cellular function of this enzyme.

## COMMENTARY

Most microorganisms, especially those that thrive in the soil, possess a range of catabolic enzymes that enable them to utilize the various substrates found on the rarely lavishly set lunch table as sources of energy and carbon. If more than one substrate is available, organisms typically utilize the one that provides the most energy first, while the synthesis of enzymes required to metabolize the less preferred substrate is suppressed to avoid unnecessary costs associated with gene transcription and subsequent translation. The underlying decision-making process is known as “carbon catabolite repression,” which has been well studied in *Escherichia coli* and *Bacillus subtilis*, but less so in other microorganisms (1). However, this mechanism is not limited to carbon-based energy sources; it also applies to energy-providing inorganic compounds, such as molecular hydrogen. A model organism that can grow with H_2_ as the energy source is *Cupriavidus necator*, which expresses its H_2_-oxidizing enzymes, the [NiFe]-hydrogenases, at temporarily elevated H_2_ concentrations. In this organism, hydrogenase gene expression is controlled in an H_2_-dependent manner by a specialized regulatory hydrogenase linked to a two-component regulatory system, whose response regulator acts as a transcriptional activator. However, transcription activation takes place only in the absence of carbon/energy sources preferred over H_2_ (2, 3). Here, we see the typical two-stage regulation of catabolic enzymes, which involves a specific response to the presence of the substrate (or a downstream catabolic intermediate) and an overarching mechanism of catabolite repression.

But what about catabolic enzymes whose substrate is always present in the same amount?

One interesting example is the so-called “high-affinity” [NiFe]-hydrogenases, which, unlike “conventional” [NiFe]-hydrogenases, are capable of sustaining H_2_-oxidation catalysis at very low H_2_ concentration (0.4 nM in aqueous solution), as is constantly present in the Earth’s atmosphere (4). These enzymes are widespread in soil microorganisms (encoded in the genomes of 22 prokaryotic phyla) and responsible for the annual breakdown of 70 million tons of atmospheric H_2_ (5). Although their catalytic turnover rates are intrinsically low, their H_2_ oxidation activity provides starving and dormant microorganisms with enough energy (6) to survive in the long term under extreme conditions of deprivation.

High-affinity [NiFe]-hydrogenases occur in two different phylogenetic flavors designated as Hhy and Huc. The model soil bacterium *Mycobacterium smegmatis* harbors both enzymes (7). While Huc, whose impressive structure is now available (8), has been shown to have the highest catalytic activity during the transition from growth to dormancy, Hhy supports survival during long-term dormancy. The expression of both enzymes, however, is suppressed in the presence of sufficient amounts of organic carbon sources, such as glucose, glycerol, and organic acids (9), suggesting possible regulation by carbon catabolite control.

In a recent paper in *mSystems*, Kropp and colleagues set out to identify regulatory proteins that may control the expression of the genes encoding the Huc hydrogenase in response to carbon starvation (10). In their study, they focused on Huc synthesis during growth on glycerol, which is an excellent carbon source for *M. smegmatis*. The key proteins responsible for glycerol uptake and its conversion into metabolizable dihydroxyacetone phosphate are encoded by the *glpFKD* operon, whose expression is induced by the transcription activator GylR in the presence of glycerol-3-phosphate (11). The authors observed that in a *M. smegmatis gylR* mutant, not only was the growth rate on glycerol significantly decreased, but, surprisingly, Huc activity in the exponential growth phase was more than 50 times higher than in the wild-type strain. A control experiment revealed that the *gylR* mutant grew normally on all other carbon sources tested and that the corresponding Huc activities remained as low as in the wild-type strain, indicating that GylR plays a role only during growth on glycerol. To determine whether GylR directly regulates *huc* gene expression, the authors employed the classical electrophoretic mobility shift assay, in which they incubated the purified GylR regulator with a DNA fragment containing the *huc* promoter region. While a clear interaction between GylR and the promoter region of the *glpFKD* operon was observed, no binding of GylR to the *huc* promoter was detected, regardless of whether glycerol-3-phosphate was present or not. This result led the authors to conclude that the high Huc activity during growth on glycerol is due to a regulatory effect indirectly caused by the *gylR* mutation. The underlying regulatory mechanism is likely also responsible for the significant changes in the proteome pattern of the *gylR* mutant, as revealed by shotgun proteomics analysis. Apart from a high level of Huc hydrogenase components, proteins involved in central catabolism—such as those in the Entner–Doudoroff pathway and the pentose phosphate pathway—were significantly less abundant than in the wild type, whereas components of metabolic pathways assumed to prepare the cells for dormancy were present in higher concentrations (10). All these effects are consistent with a carbon catabolite control mechanism, which is likely triggered by the poor growth on glycerol caused by the *gylR* mutation.

Interestingly, Huc activity in the *gylR* mutant was significantly higher during exponential growth on glycerol than in the stationary phase, whereas the opposite effect was observed in the wild-type strain. Furthermore, the shotgun proteomics analysis showed that the second high-affinity hydrogenase from *M. smegmatis*, Hhy, appeared to be unaffected by the *gylR* mutation. These observations show that the regulatory processes occurring during the exponential (with low growth rates) and stationary growth phases result in different synthesis rates of high-affinity hydrogenases (10). However, the underlying signals—whether they relate to the concentrations of key metabolic intermediates, the redox status, or other factors—and the associated regulatory proteins, have yet to be identified.

In summary, the expression of the genes encoding the Huc hydrogenase of *M. smegmatis* is substrate-independent since H_2_ is consistently present in an amount that can only be converted by high-affinity hydrogenases. However, the *huc* gene expression is governed by a carbon catabolite repression mechanism that prevents the energy-intensive synthesis of the enzyme when better energy sources than H_2_ are available. Thus, the H_2_-based energy conversion capacity of Huc comes into play when the cellular metabolic state becomes critical, and it appears to be precisely the state that prevails in the *gylR* mutant when growing on glycerol.

A welcome side effect of this study is that the discovery of an *M. smegmatis* mutant capable of synthesizing large amounts of the catalytically active Huc enzyme provides an attractive basis for enzyme production for biotechnological applications, such as enzymatic fuel cells that run on air alone (12).

## References

[1] Görke B, Stülke J. 2008. Carbon catabolite repression in bacteria: many ways to make the most out of nutrients. Nat Rev Microbiol 6:613–624. doi:10.1038/nrmicro1932 18628769

[2] Lenz O, Friedrich B. 1998. A novel multicomponent regulatory system mediates H_2_ sensing in Alcaligenes eutrophus. Proc Natl Acad Sci USA 95:12474–12479. doi:10.1073/pnas.95.21.12474 9770510 PMC22855

[3] Lenz O, Bernhard M, Buhrke T, Schwartz E, Friedrich B. 2002. The hydrogen-sensing apparatus in Ralstonia eutropha. J Mol Microbiol Biotechnol 4:255–262. 11931556

[4] Greening C, Constant P, Hards K, Morales SE, Oakeshott JG, Russell RJ, Taylor MC, Berney M, Conrad R, Cook GM. 2015. Atmospheric hydrogen scavenging: from enzymes to ecosystems. Appl Environ Microbiol 81:1190–1199. doi:10.1128/AEM.03364-14 25501483 PMC4309691

[5] Greening C, Grinter R. 2022. Microbial oxidation of atmospheric trace gases. Nat Rev Microbiol 20:513–528. doi:10.1038/s41579-022-00724-x 35414013

[6] Soom S, Moning SU, Cook GM, Lingford JP, Kropp A, Tran S, Grinter R, Greening C, von Ballmoos C. 2025. ATP synthesis driven by atmospheric hydrogen concentrations. Proc Natl Acad Sci USA 122:e2506353122. doi:10.1073/pnas.2506353122 40705430 PMC12318194

[7] Greening C, Berney M, Hards K, Cook GM, Conrad R. 2014. A soil actinobacterium scavenges atmospheric H_2_ using two membrane-associated, oxygen-dependent [NiFe] hydrogenases. Proc Natl Acad Sci USA 111:4257–4261. doi:10.1073/pnas.1320586111 24591586 PMC3964045

[8] Grinter R, Kropp A, Venugopal H, Senger M, Badley J, Cabotaje PR, Jia R, Duan Z, Huang P, Stripp ST, Barlow CK, Belousoff M, Shafaat HS, Cook GM, Schittenhelm RB, Vincent KA, Khalid S, Berggren G, Greening C. 2023. Structural basis for bacterial energy extraction from atmospheric hydrogen. Nature 615:541–547. doi:10.1038/s41586-023-05781-7 36890228 PMC10017518

[9] Cordero PRF, Grinter R, Hards K, Cryle MJ, Warr CG, Cook GM, Greening C. 2019. Two uptake hydrogenases differentially interact with the aerobic respiratory chain during mycobacterial growth and persistence. J Biol Chem 294:18980–18991. doi:10.1074/jbc.RA119.011076 31624148 PMC6916507

[10] Kropp A, Archer JD, Jespersen M, Watts TD, Solari J, Huang C, Schittenhelm RB, Grinter R, Greening C. 2026. Atmospheric hydrogen consumption is regulated by glycerol-mediated catabolite repression in mycobacteria. mSystems 11:e01678-25. doi:10.1128/msystems.01678-25 41837639 PMC13098282

[11] Bong H-J, Ko E-M, Song S-Y, Ko I-J, Oh J-I. 2019. Tripartite regulation of the glpFKD operon involved in glycerol catabolism by GylR, Crp, and SigF in Mycobacterium smegmatis. J Bacteriol 201:e00511-19. doi:10.1128/JB.00511-19 31570530 PMC6872199

[12] He K, Lingford JP, Wang F, Dong D, Kropp A, Grinter R, Greening C, Wang H. 2025. Nanoengineered bioanode with oxygen-insensitive hydrogenase for sustainable energy harvesting from atmospheric hydrogen and waste gases. Nano Energy 143:111358. doi:10.1016/j.nanoen.2025.111358

